# Editorial

**Published:** 1842-01-15

**Authors:** 


### THE MEDICAL EXAMINER.

PHILADELPHIA, JANUARY 15, 1842.

### *Susceptibility to Pain during Operations.*

If apology be needed for the introduction of the extra professional notes found in the department of original communications, we trust it will be found in the light which they throw upon questions continually and most anxiously propounded by our patients when about to undergo formidable or tedious operations—questions relating to the degree and character of the pain about to be endured. Very rarely have we met with evidences derived from the observation of the patients who have undergone such trials, noted at the moment and during the succeeding days with so much philosophic coolness. The susceptibility to pain varies in different persons; and, on one occasion, we were told by the patient, after a very protracted dissection for the removal of several fragments of the bones forming the elbow-joint, that he suffered no pain whatever. Cases of this character have been usually fatal in their issue; being generally, though not invariably, the result of a deficiency of functional activity in the nervous system. The late Francis Higgins, a former steward of the Pennsylvania Hospital, declared, when advanced in life, that he had never suffered pain, and knew not the sensation, inquiring,—in frank wonder,—what was the meaning of the term; although, when acting as jailor in the terrible period of yellow fever, either in 1797 or 1798, he had been wounded in the abdomen by a prisoner, in an attempt upon the gates of the city prison from within, and actually ran nearly half a square with the protruding intestines suspended in his hands, until caught by a citizen while in a frantic state of mind, but unconscious of the injury. In this case, the immunity from pain was not the result of deficient excitability of the organs of sense acting in their healthful conditions; for his senses were acute. It is a curious fact that, though peculiarly free from the painful consequences of mechanical injuries, he was not equally exempt from the indirect results of internal disease ; for, in his first and last severe illness—that which caused his death—he suffered intensely and acutely from a pulmonary affection.

---

It seems that a second edition of the old contest between Abernethy and the London Lancet is now enacting in New York. Dr. Mott, of the new medical school of that city, objects to the publication of notes of his lectures in the New York Lancet, a new medical periodical just started under the auspices of the publisher of the New York Herald. The editor of the Lancet, not yielding his purpose to make use of the professor's lectures, is excluded from the college, and a chancery injunction is issued to arrest the publication of the lectures. We suppose it is intended to refer the matter to a legal decision, and, in the mean time, both professor and journal reap the indirect advantages of the notoriety which follows such a contest. The abstract right in the question appears to us to be mainly on the side of the editor of the Lancet. The privilege of taking and using notes of a public course of lectures is included in the purchase of a ticket—in the absence, at least, of a special stipulation to the contrary. It may be that it is uncourteous and unfair to use the privilege against the wishes of a lecturer, and he may reasonably complain and seek redress, if his thoughts or language are misrepresented. Few lecturers, however, are disposed to withhold their lectures from publication; and Professors in medical schools, particularly, are in general nothing loath to the circulation of a good lecture in a medical journal. Dr. Mott specially objects, on the ground that the freshness will be taken off a similar publication of his own which he has in immediate contemplation. But the publication of a series of valuable *notes* of his lectures, would no doubt be the best advertisement for a subsequent fuller course to be issued under his own sanction. Not that we suppose, however, that the booksellers deem the hubbub of a chancery injunction and discussion in the newspapers, a bad herald of a new work.

The publication of courses of lectures has formed part of the plan of the Examiner during past years. We are now inclined so far to modify our plan, as to confine ourselves to occasional lectures, or short courses on specialities. Systematic courses, particularly when the same subjects come to be repeated by different hands, are overcharged with elementary matter, familiar to every educated physician. The London Medical Gazette and Lancet, which have in former times given a large number of sets of lectures, are now very much narrowing the space assigned to these subjects, and, in fact, appear to have adopted about the plan which we shall follow in the Examiner.

---

*Medical Students of Philadelphia.*—There seems to be a disposition in some quarters in New York, to harp upon a recent disturbance at a place of public amusement in this city, with the sinister aim of creating an unfavourable impression of the moral habits of the students of medicine who frequent Philadelphia. The intention here is too palpable to take effect. We would, moreover, suggest to the parties concerned, that they are impolitic in thus pertinaciously insulting the large and respectable body of students who frequent Philadelphia. We have no local or party feeling in the matter, and it is well known that we have not shrunk from free comment upon the professors and institutions of this city. But we deem it simple justice to expose and disprove these contemptible insinuations against the students collected here. The *émeute* which has been so grossly magnified for party purposes, was a simple quarrel at one of the theatres, into which a few medical students were drawn by very unprovoked aggression. The general deportment of the students here is unexceptionable. The great body of them are devoted to their studies; some find time for occasional relaxation, and no doubt partake of the amusements which abound in a large city; but the large mass, in gentleman-like bearing, and orderly habits, will stand comparison with any similar collection of young men. These attacks upon them from New York are easily seen through.

